# Correlation of magnetic and magnetoresistive properties of nanoporous Co/Pd thin multilayers fabricated on anodized TiO_2_ templates

**DOI:** 10.1038/s41598-020-67677-0

**Published:** 2020-07-02

**Authors:** Thi Ngoc Anh Nguyen, Julia Kasiuk, Wen-Bin Wu, Julia Fedotova, Janusz Przewoźnik, Czesław Kapusta, Olga Kupreeva, Serguei Lazarouk, Thi Thanh Hai Cao, Thi Thanh Thuy Nguyen, Hung Manh Dinh, Khanh Tung Do, Thanh Huong Nguyen, Hong Ky Vu, Dinh Lam Vu, Johan Åkerman

**Affiliations:** 1 Institute of Materials Science, Vietnam Academy of Science and Technology, 18 Hoang Quoc Viet, Cau Giay, Hanoi, Vietnam; 2 Vietnam Academy of Science and Technology, Graduate University of Science and Technology, 18 Hoang Quoc Viet, Cau Giay, Hanoi, Vietnam; 3 Institute for Nuclear Problems, Belarusian State University, 220006 Minsk, Belarus; 4 Department of Solid State Physics, Faculty of Physics and Applied Computer Science, AGH University of Science and Technology, 30-059 Krakow, Poland; 5 Belarusian State University of Informatics and Radioelectronics, 220013 Minsk, Belarus; 6 Physics Department, Hanoi National University of Education, 144 Xuan Thuy, Cau Giay, Hanoi, Vietnam; 7 Department of Physics, University of Gothenburg, 41296 Gothenburg, Sweden

**Keywords:** Materials science, Nanoscience and technology, Physics

## Abstract

In this study, we consider a technological approach to obtain a high perpendicular magnetic anisotropy of the Co/Pd multilayers deposited on nanoporous TiO_2_ templates of different types of surface morphology. It is found that the use of templates with homogeneous and smoothed surface relief, formed on silicon wafers, ensures conservation of perpendicular anisotropy of the deposited films inherent in the continuous multilayers. Also, their magnetic hardening with doubling of the coercive field is observed. However, inhomogeneous magnetic ordering is revealed in the porous films due to the occurrence of magnetically soft regions near the pore edges and/or inside the pores. Modeling of the field dependences of magnetization and electrical resistance indicates that coherent rotation is the dominant mechanism of magnetization reversal in the porous system instead of the domain-wall motion typical of the continuous multilayers, while their magnetoresistance is determined by electron-magnon scattering, similarly to the continuous counterpart. The preservation of spin waves in the porous films indicates a high uniformity of the magnetic ordering in the fabricated porous systems due to a sufficiently regular pores array introduced into the films, despite the existence of soft-magnetic regions. The results are promising for the design and fabrication of future spintronic devices.

## Introduction

The progress in information technologies and smart magnetic sensing requires magnetic data storage with ultra-high information density and media with high magnetic field sensitivity, possessing nanoscale spatial resolution. The arrays of nanodots and related nanostructures with the basic property of perpendicular magnetic anisotropy (PMA) have shown great potential to satisfy these issues^1–5^. The above-mentioned challenges require advanced but inexpensive technologies for producing high-quality, large-area arrays of nanostructures with stable and well-reproducible magnetic properties. The main methods commonly used in fabricating nanostructures are high resolution lithographic technologies, such as electron-beam and X-ray lithography^2,6–8^. However, these techniques have essential shortcomings associated with the extremely high costs, low throughput and small exposure area. Besides the technical issues, the main physical limitations hindering the progress in these technologies are set by thermal instability of magnetically-decoupled superparamagnetic (SP) nanostructures^2,3^ since the size of isolated single-domain magnetic islands containing each bit of information is required to be persistently reduced. One approach to delaying the onset of the SP limit is to improve the magnetic anisotropy of nanostructures exploited in bit patterned media (BPM)^8^. The other approach is based on the principally different materials, percolated perpendicular media (PPM)^4,5^, containing a continual perpendicularly-anisotropic film with a dense distribution of defects (nanoholes/antidots) which serve as domain-wall pinning sites^9,10^. Intensive research in recent decades has shown that the PPM concept works well with magnetically coupled 3*d*/5*d* multilayers (MLs), such as Co/Pd and Co/Pt, which are deposited on self-organized nanoporous templates (Al_2_O_3_, TiO_2_)^1,5,11–13^. In the PPM approach, the implemented nanopatterning of a continuous magnetic film is accompanied by conservation of its high PMA, while preserved exchange coupling provides the magnetic stability^9^ of nanostructures.

Magnetic anisotropy of such nanoporous films demonstrates high sensitivity to the morphological features of templates used as a substrate^5,11,14^. It was shown that a well-developed morphology of nanoporous films with numerous inhomogeneities results in a pronounced deterioration of PMA due to the local misalignments of easy magnetization axes at the edges of nanopores^15^. At the same time, despite an abundant amount of data on the continuous Co/Pd and Co/Pt MLs^16–18^ accumulated since 1988^19^, only a few studies were devoted to finding out the correlation between the surface morphology of the corresponding nanostructured films and the strength of their magnetic anisotropy^5,11–14,20,21^. Therefore, the present research is focused on elucidating the influence of the morphology of the Co/Pd multilayered films, related to the used TiO_2_ templates, on their magnetic parameters (coercive field *H*_C_, remanent magnetization *M*_r_, etc.) which, in turn, determine their potential functionality. The main aspect of the study is a detailed analysis and interpretation of magnetization reversal mechanisms characterizing porous Co/Pd films with strong PMA. The processes of magnetization reversal in the films were modeled within the frame of the Stoner–Wohlfarth model^15,21,22^ and analyzed in relation to the morphology of the films, as well as their phase composition.

Having regard to the high potential of anisotropic magnetic media for spintronics applications, the spin-dependent electronic transport in the studied MLs is analyzed in detail for determining the magnetoresistance (MR) mechanisms and elucidating the influence of the magnetic ordering and magnetization reversal in the porous Co/Pd films with the specific morphology on their MR signal. A model interpretation of the field dependences of MR was carried out for both continuous and porous multilayered systems with the assumption of electron-magnon scattering as the dominant MR mechanism^23^.

## Results and discussion

### Scanning electron microscopy

**Figure 1:**
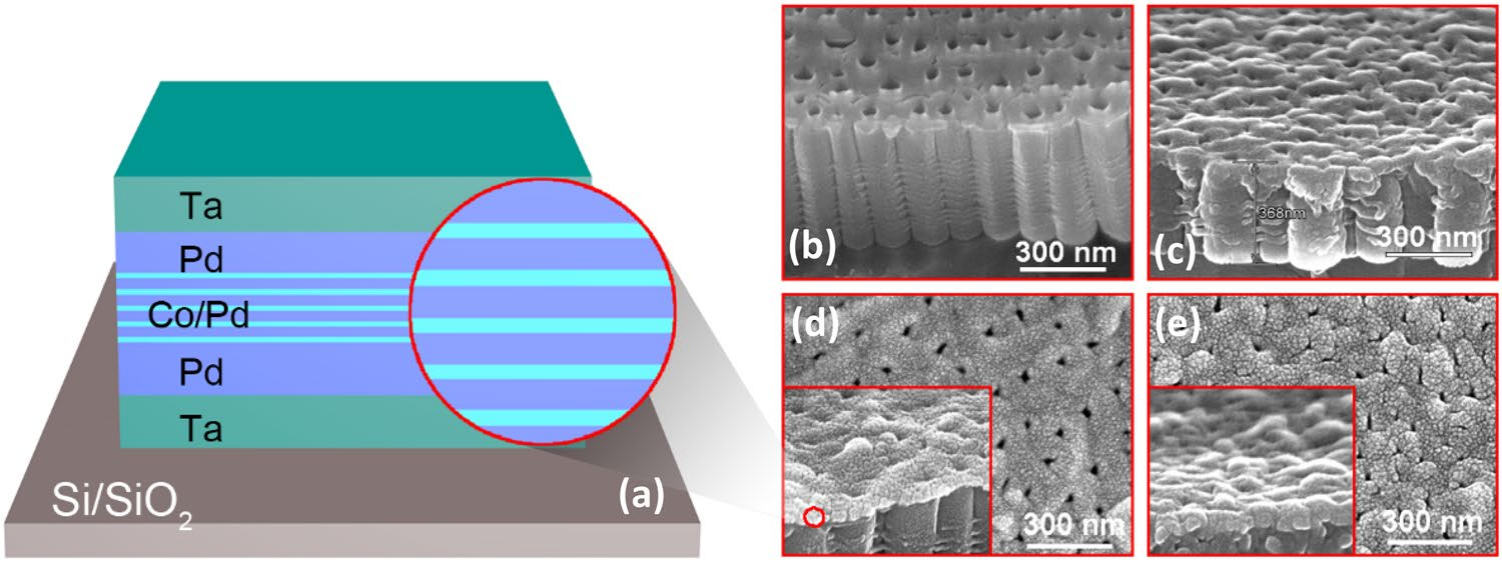
(**a**) Schematic representation of the composition of the deposited Co/Pd films. (**b**, **c**) SEM images of TiO_2_ templates fabricated on (**b**) Ti foil and (**c**) Si wafer. (**d**, **e**) SEM images of the Co/Pd MLs deposited on (**d**) Ti/TiO_2_ and (**e**) Si/TiO_2_ templates.

A schematic representation of the composition of the studied Co/Pd multilayered films illustrating the sequence of their layers is shown in Fig. 1a. Typical images of scanning electron microscopy (SEM) demonstrating surface morphology of the nanoporous TiO_2_ templates used for Co/Pd films deposition, which are fabricated over Ti foil (Ti/TiO_2_) and Si wafer (Si/TiO_2_), are presented in Fig. 1b, c, respectively. The images are taken at an angle of 45° with respect to the surface of the templates for better surface relief illustration.

As can be derived from SEM images, the pore diameter mainly varies between 40 and 60 nm (some even up to 100 nm) for the Ti/TiO_2_ template (Fig. 1b), whereas it amounts to 20–40 nm only for the Si/TiO_2_ template (Fig. 1c). The distance between the pore centers in the Ti/TiO_2_ and Si/TiO_2_ templates is about 150 and 130 nm, respectively. The difference in the pore diameters between the two types of templates can be associated with the significantly different thermal conductivity of Si (150 W/mK) and Ti (14–22 W/mK) materials providing better heat dissipation in the case of Si substrate and, consequently, lower temperature during anodization process of Ti film. Therefore, this leads to a lower rate of chemical etching of TiO_2_ nanotubes walls within the Si/TiO_2_ template^24^. It is worth noting that these two types of templates reveal a significantly different surface relief. The Ti/TiO_2_ template demonstrates rather flat inter-pore regions and a wavy surface of the whole template, possibly because of its soft and flexible background formed by Ti foil (Fig. 1b). In contrast, the Si/TiO_2_ template has a rather homogeneous surface relief, but it contains some undesirable surface peculiarities which are visible as “hills” over the whole template surface, increasing significantly its surface roughness (Fig. 1c).

SEM images of the Co/Pd MLs fabricated on the Ti/TiO_2_ and Si/TiO_2_ templates are presented in Fig. 1d, e. They evidence the porous structure of both films, with the average pore diameter of about 35–40 nm for the MLs on the Ti/TiO_2_ template and 30–35 nm for the film on the Si/TiO_2_ template. The decrease in the pore size for the films, as compared to the initial templates, indicates that the pores are partially occluded by the deposited film^25^. Generally, the morphology of Co/Pd MLs reproduces well the template morphology (Fig. 1b, c). However, the deposited film smoothens partly the template surface inhomogeneities like small triangular pores between TiO_2_ nanotubes (Fig. 1b).

### X-ray diffraction

**Figure 2:**
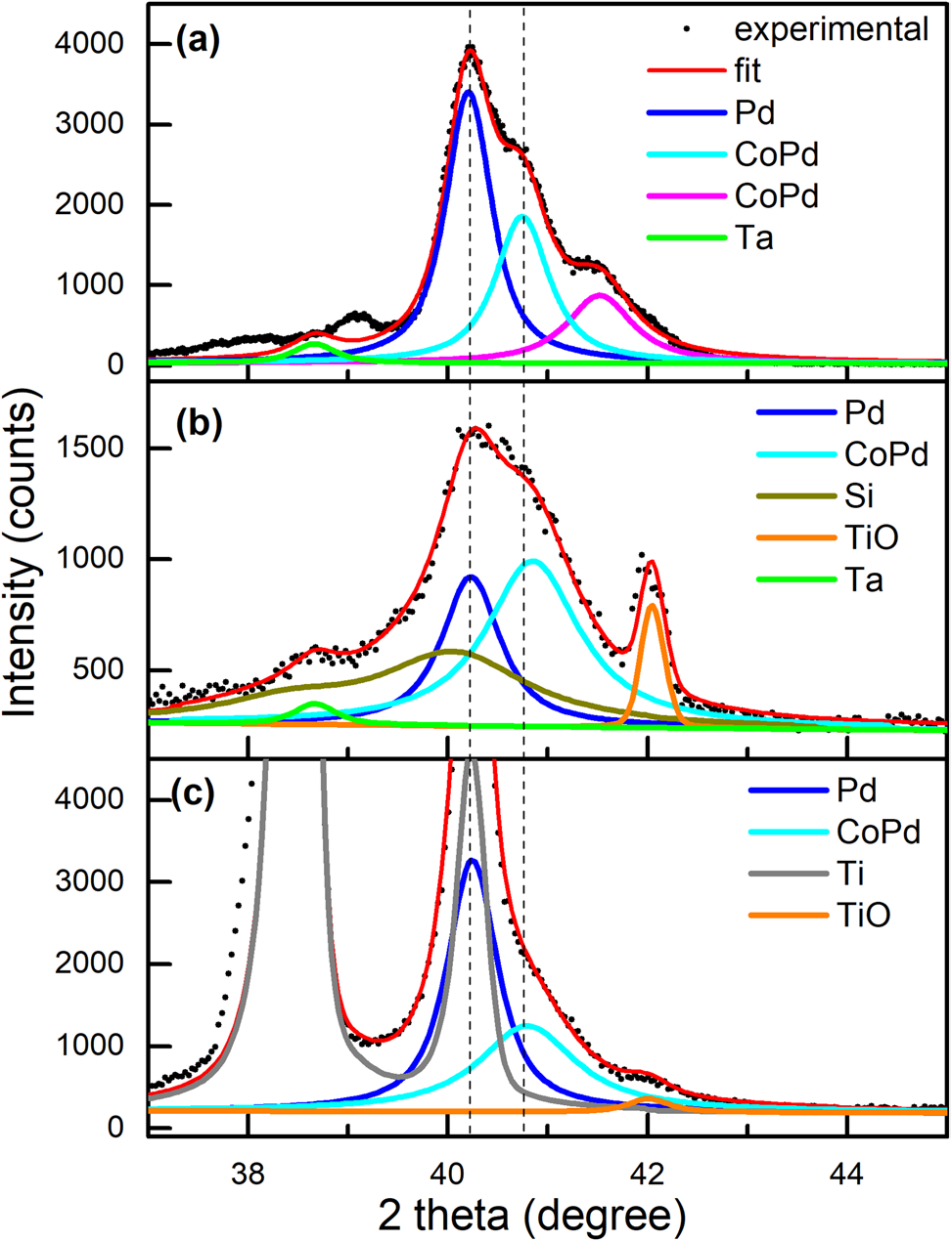
Parts of the experimental (black points) XRD patterns of the Co/Pd multilayered films on (**a**) flat Si wafer, (**b**) porous Si/TiO_2_ template and (**c**) porous Ti/TiO_2_ template, together with the results of their refinement (lines).

Experimental X-ray diffraction (XRD) patterns both for the continuous (reference sample) and porous Co/Pd MLs are presented in Fig. 2. They are accompanied by the results of their refinement and partial phase contributions evaluated from the fits. The parameters describing crystalline structure of the phases detected in the Co/Pd MLs, which are extracted from the refinement of their XRD patterns, are collected in Table 1.

**Table 1:** Parameters describing the crystalline structure of the phases detected in the Co/Pd MLs fabricated on a flat Si wafer, as well as on the porous Si/TiO_2_ and Ti/TiO_2_ templates, which are extracted from the refinement of their XRD patterns (the main peak positions 2θ on the XRD patterns and the corresponding lattice parameter *a*).

Substrate	Phase	2θ, deg	*a*, Å
Si wafer	Pd	40.18	3.884
	CoPd	40.72	3.835
	CoPd	41.49	3.766
Si/TiO_2_ template	Pd	40.20	3.882
	CoPd	40.82	3.826
Ti/TiO_2_ template	Pd	40.21	3.881
	CoPd	40.77	3.831

As shown in Fig. 2a, the continuous Co/Pd film demonstrates three main overlapping diffraction peaks at 2θ ~ 40.18°, 40.72° and 41.49°. The dominant line at 2θ ~ 40.18° corresponds well to the position of the main (111) peak of a face-centered (*fcc*) structure of Pd with *a* = 3.884 Å^26^ and originates mainly from the buffer and capping Pd layers. Two additional peaks at 2θ ~ 40.72° and 41.49° are associated with two phases of *fcc* CoPd alloy with slightly different lattice parameters *a* = 3.835 Å and 3.766 Å appearing as a result of commonly observed mixing at the interface between Co and Pd layers. The presence of two modifications of CoPd alloy in the film indicates that Co concentration^26^ in MLs varies with depth. Based on the *a* value, the stoichiometry of the CoPd alloys corresponding to the 2θ = 40.72° and 41.49° peaks (Table 1) is estimated to be Co_26_Pd_74_ and Co_45_Pd_55_, respectively^15,26^.

XRD analysis of the porous Co/Pd MLs on the Si/TiO_2_ template (Fig. 2b) reveals that the structural parameters of the Pd and CoPd phases reproduce well those for the continuous film (Table 1). The only new aspect, a relatively narrow peak from TiO structure (*Fm3m*, *a* = 4.297 Å)^27^ appears in the XRD patterns of the porous films originating from the template, with its main TiO_2_ phase being amorphous. Additionally, the only peak characterizing CoPd alloy is detected in the porous films, which demonstrates a comparable intensity with the peak of pure Pd (Fig. 2b). These facts indicate a substantial Co and Pd mixing in the porous MLs. In addition, a more uniform mixing in the porous Co/Pd MLs can be stated, as compared to the corresponding flat system. The value of *a* parameter obtained for the CoPd alloy in the porous Co/Pd MLs (Table 1) allows us to estimate its stoichiometry as Co_30_Pd_70_. The obtained atomic ratio is close to the composition of the CoPd_2_ phase^15,28^, which, in turn, corresponds to the ratio of the thicknesses of the deposited Co and Pd layers. This fact indicates a complete mixing of Co and Pd layers inside the Co/Pd MLs that is in agreement with the high tendency to Co and Pd atom mixing ^15,16,29^.

The XRD pattern of the porous Co/Pd MLs on the Ti/TiO_2_ template is presented in Fig. 2c. Two intense peaks at 2θ ~ 38.4° and 40.2° correspond to the Ti phase of the substrate. The refinement of the XRD pattern allowed revealing the Pd and CoPd phases after the Ti peaks identification. In this way, the extracted lattice parameters (Table 1) are in a good agreement with the corresponding parameters of bulk *fcc* Pd (*a* = 3.890 Å^12^) and *fcc* CoPd_2_ (*a* = 3.830 Å^8,13^) phases, although the latter is not supposed to be well-ordered in our films, because the deposition was performed at room temperature. As can be seen in Fig. 2, the diffraction peaks of the CoPd alloy are broadened, especially in the case of the porous MLs, which confirms its poor crystallinity.

### Room-temperature magnetometry

**Figure 3:**
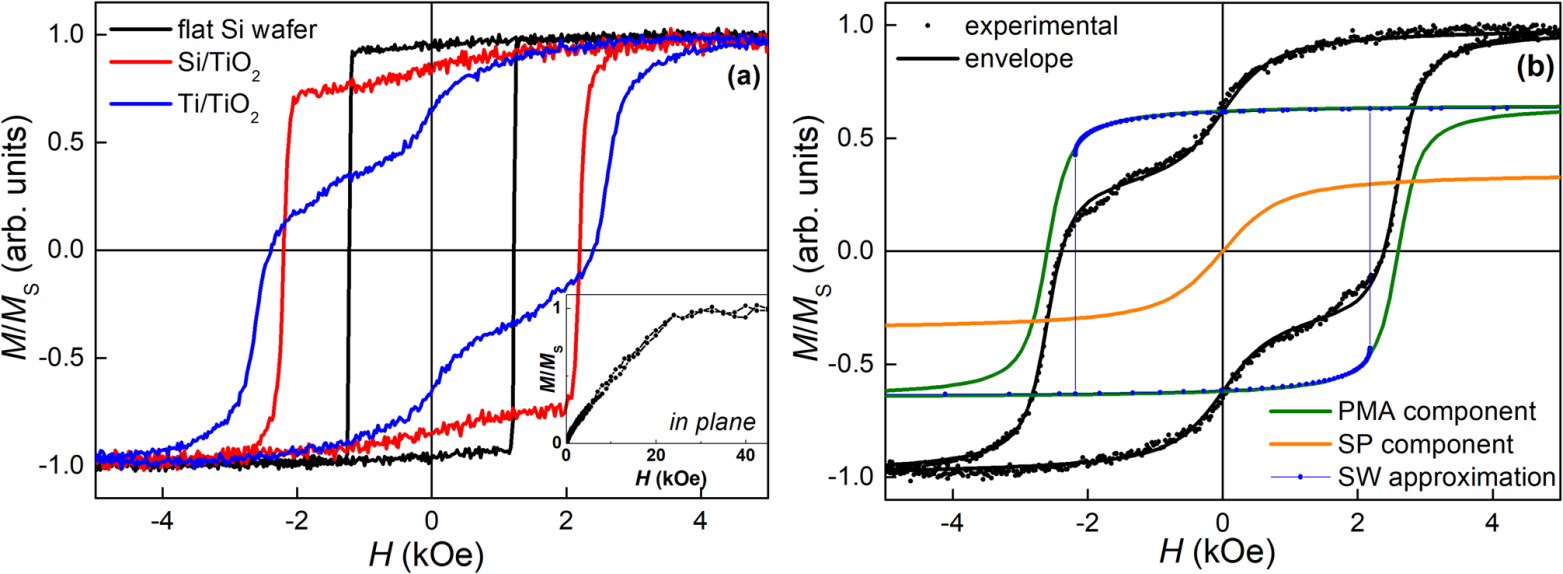
(**a**) Experimental RT magnetization curves *M*(*H*)/*M*_S_ of the Co/Pd MLs on a flat Si wafer and on the porous Si/TiO_2_ and Ti/TiO_2_ templates measured in the film normal direction. The inset shows the *M*(*H*)/*M*_S_ curve of the continuous Co/Pd MLs on a flat Si wafer obtained in the film plane. (**b**) Approximation of the RT magnetization curve *M*(*H*)/*M*_S_ of the porous Co/Pd MLs on Ti/TiO_2_ template measured in the film normal direction, together with the extracted components of hard- and soft-magnetic phases; the hard-magnetic component (solid green line) is fitted with the Stoner–Wohlfarth (SW) model whereas the soft-magnetic one (solid red line) is modeled with the Langevin function.

Figure 3 represents the experimental field dependences of the normalized magnetization *M*(*H*)/*M*_S_ (where *M*_S_ is the saturation magnetization value) obtained for the continuous and nanoporous Co/Pd MLs at room temperature (RT) in a magnetic field *H* applied along the normal to the film surface. The magnetization curves of the continuous film demonstrate high magnetic squareness ratio *M*_*r*_/*M*_S_ ~ 0.96. This observation, together with a relatively high coercive field *H*_C_ in this direction of about 1.2 kOe, indicates that the Co/Pd MLs are characterized by a pronounced PMA^13,15^. An anisotropy field *H*_A_ of the MLs can be estimated from the saturation of their magnetization in the hard direction, i.e. along the film plane. The corresponding *M*(*H*)/*M*_S_ curve of the continuous Co/Pd MLs is shown in the inset to Fig. 3. As it can be seen, the in-plane magnetization curve of this film is characterized by a rather simple shape with a linear increase in *M* up to saturation and the *H*_A_ field close to 24.5 kOe. The obtained *H*_A_ value of the studied MLs reaching 35 kOe at *T* = 50 K is comparable with the maximal achieved anisotropy fields for similar Co/Pd and Co/Pt multilayered films with strong PMA (*H*_A_ ~ 15–30 kOe)^15,18,30,31^. High *H*_A_ value of the studied Co/Pd MLs is supposed to determine mainly the peculiarities of their magnetic and magnetoresistive properties described below.

The RT magnetization curves of the porous Co/Pd MLs reveal a gradual decrease in their *M*_r_/*M*_S_ parameter (see Fig. 3a) when going from Si/TiO_2_ to Ti/TiO_2_ template. Actually, using the porous Si/TiO_2_ template for the film deposition leads to a slight decrease in its *M*_r_/*M*_S_ value down to 0.85. Next, using the porous Ti/TiO_2_ template on a flexible Ti foil leads to a further significant decrease in the *M*_r_/*M*_S_ ratio (down to 0.65) of the deposited Co/Pd MLs that indicates a dramatic decrease in their PMA effect. The latter shows that microscale imperfections and a wavy surface, which are typical of the templates on foils, deteriorate PMA of the deposited films more substantially than local nano-sized inhomogeneities of the Si/TiO_2_ templates forming their developed relief (Fig. 1c).

It is worth noting that a huge increase in *H*_C_ up to 2.4 kOe is detected for the porous Co/Pd films as compared to their continuous counterpart. Remarkably, unusual double-step magnetization curves are observed for the porous films, which are characterized by two distinct steps, with the steps being more pronounced for the film on the Ti/TiO_2_ template (Fig. 3). The latter indicates inhomogeneity of magnetic ordering in the films, i.e. the coexistence of the regions with significantly different magnetic hardness and, possibly, obeying different mechanisms of magnetization reversal.

An analysis of the possible mechanisms of magnetization reversal in the Co/Pd MLs demonstrating complex *M*(*H*)/*M*_S_ dependence was carried out. For this purpose, the magnetization curve of the porous Co/Pd MLs on Ti/TiO_2_ template is decomposed into two components describing each step separately^32,33^, as it is shown in Fig. 3b. As can be seen from the figure, one of the components is the loop with high *H*_C_ and *M*_r_/*M*_S_ values that corresponds to the regions of the films with perfect PMA and hard-magnetic properties. The second component relates to near-edge soft-magnetic or possibly superparamagnetic (SP) material^32^ according to its nearly zero *H*_C_ value. The presence of this component decreases the total squareness *M*_r_/*M*_S_ parameter of the experimental magnetization curve, thus decreasing dramatically its PMA. The experimental curve is fitted then by the sum:

1 $$M(H)/{M}_{S}=n\times {M}_{hard}\left(H\right)+(1-n)\times {M}_{soft}(H),$$

where *M*_hard_(*H*) is the normalized magnetization curve describing the part of the film with uniaxial magnetic anisotropy, i.e. obeying the Stoner–Wohlfarth mechanism of magnetization reversal^13,15^, and *M*_soft_(*H*) is the component describing the soft-magnetic (or SP) part of the film where magnetization is governed by the Langevin function^33^. The estimated relative contribution of the soft-magnetic material, (1 − *n*), in the total magnetization makes up 35%.

The soft-magnetic (or SP) component in the magnetization of the porous films possibly originates from the structures which are formed at the pore edges and/or inside the pores. In the first case, small toroid-like nanostructures formed around the pores or spherical inhomogeneities at the film surface (Fig. 1d, e) contain misaligned magnetic moments. These moments are forced by high PMA of the film to be oriented perpendicularly to the curved surface^34^, i.e. divergently, thus forming isotropic magnetic properties of the corresponding film regions^32,34^. The second possibility assumes penetration of Co/Pd material inside the pores during deposition^5,20^. The large pore diameter (up to 100 nm for some pores) in the case of Ti/TiO_2_ templates and rather short length of TiO_2_ nanotubes (Fig. 1b) makes it possible for the studied films. Structurally detached nanodots possibly formed inside the pores can reveal a SP behavior at RT and contribute to the soft-magnetic component. After subtracting the soft-magnetic component from the total *M*(*H*)/*M*_S_ dependence, it is evident that the major part of the film demonstrates perfect PMA with the high *M*_r_/*M*_S_ ratio of 0.95 (Fig. 3b), close to that of the continuous Co/Pd MLs. Approximation of the *M*_hard_(*H*) component of the magnetization curve within the frame of the Stoner–Wohlfarth (SW) model^13,15^ is presented in Fig. 3b. It allows estimating the mean deviation α of the magnetic moments from the film normal that amounts to 15º for the Co/Pd MLs on the Ti/TiO_2_ template.

### Low-temperature magnetometry

**Figure 4:**
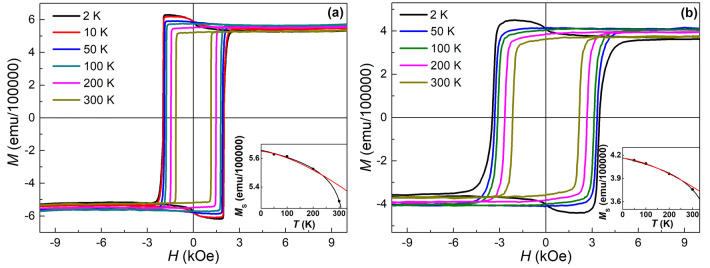
Experimental *M*(*H*) magnetization curves measured at *T* = 2–300 K in the external field *H* applied along the film normal for (**a**) continuous and (**b**) porous Co/Pd MLs deposited on Si wafer and Si/TiO_2_ template, respectively. The insets present the temperature dependences of the saturation magnetization *M*_S_ obtained at 10 kOe for the studied MLs (points correspond to the experimental data, red line shows the approximation with the conventional Bloch’s law, and black line refers to the “modified” Bloch’s law, see the description in the text).

The experimental *M*(*H*) curves of the Co/Pd MLs on both flat Si wafer and porous Si/TiO_2_ template measured in the range of temperatures *T* = 2–300 K are shown in Fig. 4. The continuous Co/Pd MLs demonstrate a significant increase in *H*_C_ with decreasing temperature achieving 2 kOe at 2 K that is almost two times higher than its value at RT. Additionally, a small increase in the saturation magnetization can be noticed with decreasing temperature down to 50 K. Such changes in *H*_C_ and *M*_S_ parameters with lowering temperature are associated mainly with the decrement of the thermally activated magnon population, as well as an additional contribution from the magnetic polarization of Pd at low temperature^35–37^. Indeed, Pd magnetic polarization due to the adjacent Co layers goes deeply into Pd layers at low temperature which gives an additional magnetic moment (~ 0.3 µB as calculated per Pd atom^38^) and promotes better interlayer coupling^35,36^, thus enhancing PMA of the MLs at low *T*^35^. The strictly uniform orientation of magnetic moments in the film due to their reduced thermal fluctuations^36^ and an enhanced PMA^35,39^ at low temperature, as well as their strong ferromagnetic coupling^35,36^, hinders the process of magnetization reversal, increasing therefore the *H*_C_ parameter. Further temperature lowering leads surprisingly to *M*_S_ decrease down to a value close to the one observed at RT. In addition, small upward step appears on the *M*(*H*) curves at *T* < 50 K when *H* changes sign, i.e. in the vicinity of zero field. The origin of this phenomenon may be in a specific arrangement of a part of magnetic moments inside the MLs at low temperature, possibly related to Dzyaloshinskii–Moriya interaction or antiparallel ordering of Co and Pd magnetic moments, but it needs further studies. However, to our knowledge, such steps were not previously reported for similar systems in literature sources.

The temperature dependence of *M*_S_ for the continuous MLs studied is shown in the inset to Fig. 4a. It follows well the empirical expression^35,36,40^ describing thermal fluctuation of magnetization *M*_S_(*T*) = *M*_S_(0)(1 − *T*/*T*_b_)^*b*^, where *M*_S_(0) is the saturation magnetization in zero field and *T*_b_ is the blocking temperature (black line in the insets to Fig. 4). The conventional Bloch’s law *M*_S_(*T*) = *M*_S_(0)(1 − *BT*^3/2^), where *B* is the Bloch constant, implying spin excitations at nonzero temperatures as the origin of a high-temperature *M*_S_ decrease, gives a worse correspondence with the experimental data, with a strong deviation at *T* = 300 K (red line in the insets to Fig. 4). As the Bloch’s law is derived for bulk ferromagnets, it is not fully applicable to nano-scale materials due to finite size effect^40^, since interfaces prevent free propagation of spin waves with a wavelength larger than the layer thickness. Therefore, the detected deviation of the approximation with the Bloch’s law from the experimental data at RT can possibly be associated with the excitation of magnons with the energies higher than those implied by the Bloch’s law (just with the quadratic dispersion law), as well as with magnons interaction at high temperatures. It should be mentioned that the parameter *b* obtained from the approximation using the empirical expression also shows its value of 0.025, much lower than the one obtained for similar systems (*b* = 0.34)^35,36^, indicating a weak temperature dependence of *M*_S_.

The *M*(*H*) curves of the porous Co/Pd MLs (Fig. 4b) demonstrate the tendencies for their *H*_C_ and *M*_S_ parameters with lowering temperature similar to their continuous counterpart, namely, a 60% increase in *H*_C_ achieving 3.5 kOe at 2 K, as well as a 10% growth of *M*_S_ at 50 K compared to the corresponding RT values. The constant *B* estimated from the approximation of the *M*_S_(*T*) dependences according to the Bloch’s law is revealed to be significantly higher for the porous MLs (1.7 × 10^–5^ K^−3/2^) than for the continuous counterpart (0.8 × 10^–5^ K^−3/2^). The increased Bloch constant, which is formulated as *B* ~ *A*^−3/2^ (*A* is a ferromagnetic exchange stiffness constant)^41^ indicates a weakened ferromagnetic interaction between spins in the porous MLs due to the edge effects or changes in spin waves propagation caused by the complex morphology of the porous system.

It should be mentioned that in addition to an increase in *M*_S_, a gradual increase in *M*_*r*_/*M*_S_ ratio takes place with decreasing temperature for the porous MLs, which smears out the double-step shape of magnetization loops of the porous films observed at RT (Fig. 3a). Such an increase in *M*_*r*_/*M*_S_ ratio indicates (1) freezing of temperature induced fluctuations of magnetic moments in the soft-magnetic phase, assuming that it is formed by SP nanodots, or (2) strengthening the exchange coupling in ferromagnetic film at low temperature^39^, i.e. between hard and soft-magnetic phases, if the soft-magnetic phase is supposed to be formed on rough areas of the templates having high surface curvature.

### Magnetoresistance

**Figure 5:**
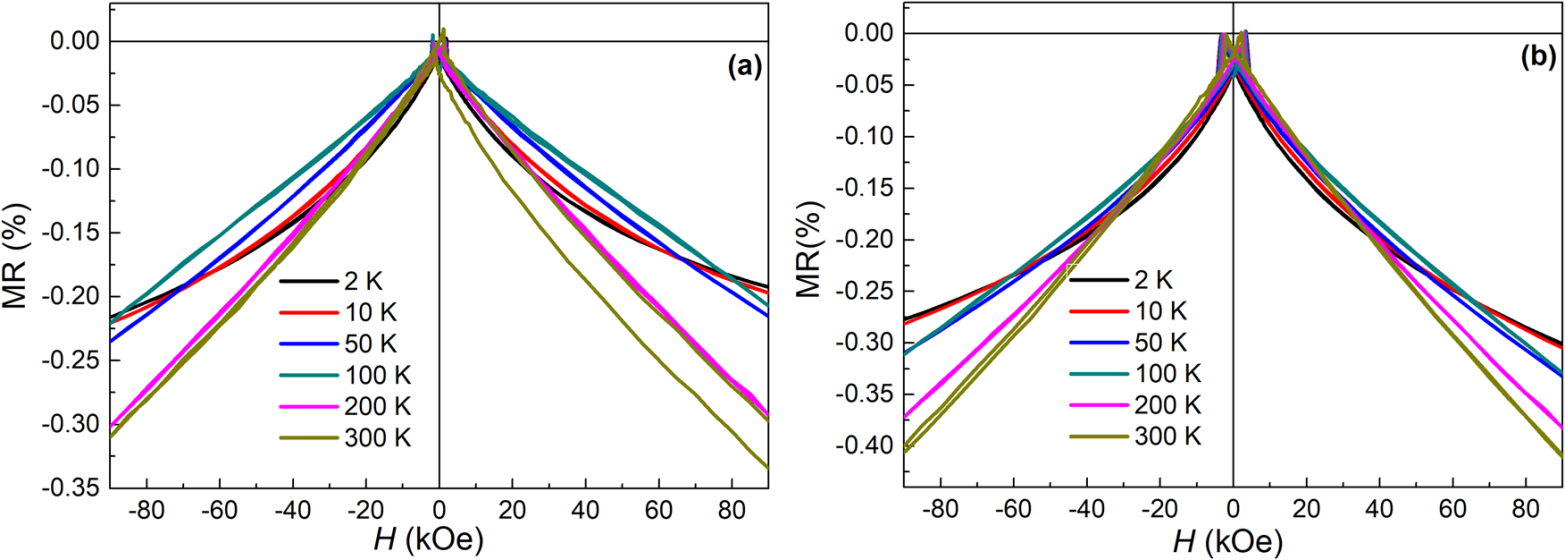
Experimental field dependences of magnetoresistance MR(*H*) measured at different temperatures in the field *H* applied along the film normal direction for (**a**) continuous and (**b**) porous Co/Pd MLs deposited on Si wafer and Si/TiO_2_ template, respectively.

The field dependences of magnetoresistance MR(*H*) = 100% × (*R*(*H*) − *R*(0))*/R*(0) of the studied Co/Pd MLs derived from the measured field dependences of their electrical resistance *R* are shown in Fig. 5 for the continuous and porous (on Si/TiO_2_ template) films at different temperatures (*T* = 2–300 K). The almost linear, unsaturated MR(*H*) dependence up to high fields (9 T) is the most evident characteristic of both continuous and porous Co/Pd MLs. Despite the metallic properties of each layer in the films, the MLs demonstrate a negative MR that indicates spin-dependent electron transport, with the effect of negative MR being enhanced with increasing temperature. In ferromagnetic metals with strongly coupled spins, their collective excitations (magnons) impede the electric current, initiating the electrons scattering on these excitations, i.e. on magnons^23,42^. Since the external field *H* tends to align misoriented magnetic moments, as well as to decrease the magnitude of their precession, it decreases the number of scattering events, thus reducing the electrical resistance. It is worth noting that such an almost linear reduction of the resistance in high fields (Fig. 5) corresponds to the saturated magnetization of the MLs (Fig. 4). This reveals a minor role of the static effects related to the magnetic moments misalignment in the described negative MR effect, giving preference to the dynamic effect of magnons. The observed decrease in the slope angle of the MR(*H*) curves to the field axis with lowering temperature associated with a decrease in the negative MR effect originates from a partial change in the MR mechanism. The contribution of magnon magnetoresistance (MMR) decreases with decreasing temperature, namely because magnons are less populated at low *T*^23,42^. On the other hand, the contribution of the positive MR via Lorenz mechanism increases with decreasing temperature due to an increase in the mean free path of electrons. The latter contribution reduces the total MR effect and diminishes the linearity of MR(*H*) curves at low temperatures.

It should be mentioned that the revealed MMR mechanism, which is associated with a damping of the spin waves in high fields (HF)^42^, is not typical of thin multilayered films similar to the studied Co/Pd MLs, since interfaces prevent the free propagation of spin waves. In doing so, such films with a small thickness of ferromagnetic layers (less than 1 nm) commonly demonstrate the saturated MR(*H*) dependences in high fields (HF-MR) corresponding to their saturated magnetization^30,43,44^. The existence of spin waves in the studied films is consistent with the mixing of their Co and Pd layers revealed by XRD, which results in the formation of ferromagnetic alloy^29,33^ with a thickness of the corresponding layer comparable to that in the systems where magnons are typically detected (no less than 7–10 nm for different 3*d* metals^23,42,45^).

One more peculiarity of the MR(*H*) curves observed at low temperatures (*T* = 2–10 K) is a faster decrease in the MR value with increasing field than predicted by the MMR model^23,42^. As a result, the MR(*H*) curve obtained at 2 K lies below the corresponding curve measured at 100 K (Fig. 5). This can be explained solely by the appearance of an additional contribution to the spin-dependent scattering of electrons at low *T*, enhancing negative MR. The latter correlates with the detected upward steps on the corresponding *M*(*H*) curves in the same temperature range (Fig. 4). Both observations are possibly associated with the opposite orientation of a part of magnetic moments in the MLs with respect to the external field. However, the mechanism of such an arrangement is still not fully understood.

**Figure 6:**
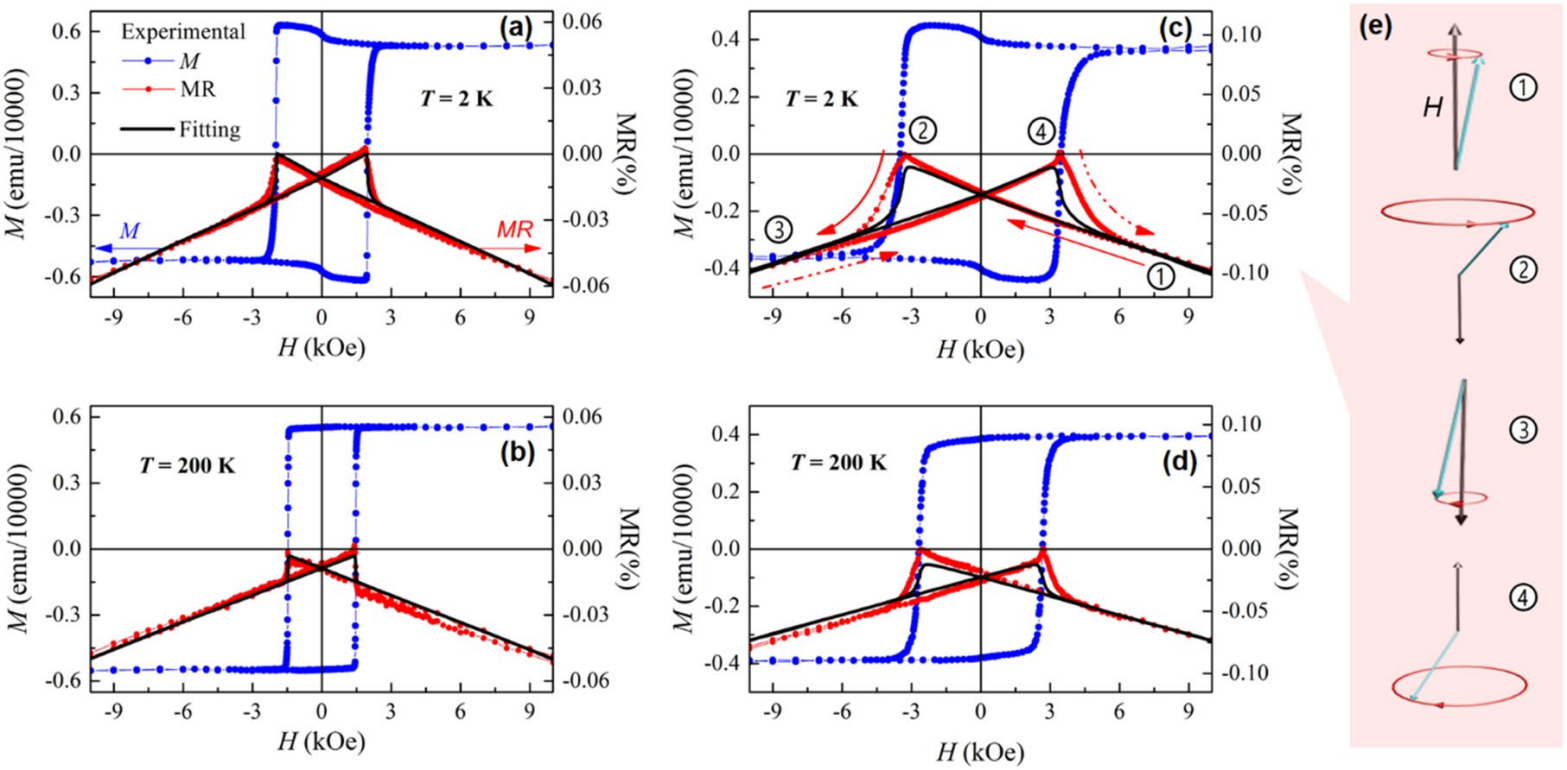
(**a**–**d**) A comparison of the experimental field dependences of magnetization *M*(*H*) (blue dots) and magnetoresistance MR(*H*) (red dots) of the Co/Pd MLs: (**a**, **b**) continuous MLs on Si wafer and (**c**, **d**) porous MLs on Si/TiO_2_ template, which are measured in a magnetic field applied along the film normal at different temperatures: (**a**, **c**) *T* = 2 K and (**b**, **d**) *T* = 200 K. Black solid lines represent the approximation of MR(*H*) dependences using Eq. (2). (**e**) Schematic illustration of rotation (precession) of magnetic moment µ (green arrow) depending on its orientation with respect to the external field *H.*

The low-field part of the MR(*H*) curves (LF-MR) has more complicated shape than a simply linear HF-MR. An evident correlation with the corresponding *M*(*H*) curves is characteristic of the LF-MR of both continuous and porous Co/Pd MLs. Figure 6 illustrates such a correlation for these two films at *T* = 2 K and 200 K. As it can be seen from the figure, a nearly linear increase in MR with decreasing field (follow the red solid arrows in Fig. 6c) is replaced by its abrupt decrease in the negative field coinciding with the coercive field of the corresponding *M*(*H*) curve. In terms of spin excitations, a decrease in a magnetic field, which suppresses spin waves, leads to an increase in the amplitude of spin precession, thus raising the electrical resistance of the film (corresponds to a linear MR increase between points 1 and 2 in Fig. 6c, or, in other words, relates to a decrease in the absolute value of MR). Then, negative external field, i.e. the field applied in the direction opposite to the orientation of magnetic moments (point 2 in Fig. 6c, e) tends to destabilize them^23^, increasing further the amplitude of their precession until their reversal. The latter corresponds to the maximal resistance of the film. Next, switching the magnetization at *H* = − *H*_C_ to the opposite direction provides parallel orientation of the magnetic moments to the external field (point 3 in Fig. 6c, e) that decreases rapidly the magnon population^23^, thus leading to a steeply diminishing resistance. Noteworthy, the amplitude of MR drop is proportional to the *H*_C_ value^23^ (Fig. 6). A further increase in a negative field provides an additional gradual decrease in the resistance due to spin waves damping in the HF region.

The approximation of the LF-MR curves of the Co/Pd MLs can be made using their *M*(*H*) dependences according to the relation proposed for the MMR mechanism^23^:

2 $$MR\left(H\right)=-\frac{M}{{M}_{\mathrm{S}}}\times\alpha \left(T\right)\times H,$$

where *α*(*T*) is a temperature-dependent slope of MR(*H*) curve in the vicinity of zero field. The results of approximation are also presented in Fig. 6 (black solid lines). A perfect coincidence of the experimental and approximating MR(*H*) curves for the continuous Co/Pd MLs (Fig. 6a, b) proves that their spin-dependent MR is realized through the electron-magnon scattering mechanism. The experimental LF-MR curves of the porous Co/Pd MLs also demonstrate a good agreement with the corresponding approximation (Fig. 6c, d). Their main discrepancy lies in a delay of the MR signal drop (red dots) with respect to the reversal of magnetic moments at *H* = *H*_C_ (blue dots and black line), with the latter process being more abrupt and nonlinear. Such a discrepancy relates to a complex origin of the MR signal. First of all, similarly to the magnetometry results, it reproduces static processes involving the magnetic moments, such as their misalignments, canting and reversal, which are reflected in the magnetization curves. However, it reveals also the sensitivity to high-frequency excitations of spins, with the corresponding MR signal being almost linear with the applied field^23^, since the number of excited or suppressed magnons is proportional to the *H* value. The latter contribution increases the linearity of the MR(*H*) curves.

Importantly, the porous structure and complex relief does not prevent spin waves propagation in the studied Co/Pd MLs. Indeed, they contain quite large inter-pore regions of tens of nanometers in length and demonstrate higher uniformity of Co and Pd mixing than the continuous film, according to the XRD data (Fig. 2). In addition, a significantly lager effect of negative MR is characteristic of the porous Co/Pd MLs as compared to the continuous counterparts (almost two-fold difference in *H* = 10 kOe, Fig. 6a, c). This can even indicate more intense spin waves in the porous system, which correlates with its larger Bloch constant estimated from the magnetization curves.

## Conclusions

We have carried out a detailed analysis of the role of surface morphology in magnetically ordered Co/Pd MLs with pronounced PMA (*H*_A_ = 25 kOe) deposited onto the porous Ti/TiO_2_ and Si/TiO_2_ templates with significantly different surface relief. The morphology of the Co/Pd films is found to reflect the features of the surface of the templates used. As a result, a more homogeneous and smoothed relief is characteristic of the film deposited on the Si/TiO_2_ template that provides a distinct PMA with high *M*_r_/*M*_S_ ratio reaching 0.85 at RT. The film on the Ti/TiO_2_ template contains pores with larger diameter (~ 40 nm) and microscale surface imperfections and undulations, which are responsible for the inhomogeneous magnetic ordering in the film and the occurrence of a soft-magnetic or superparamagnetic component. This component of the film is formed on the areas of the template with high surface curvature, like pore edges and surface convexities, and/or inside the pores. This partly deteriorates the PMA of the film, decreasing its *M*_r_/*M*_S_ ratio down to 0.65, but provides the maximal coercive field *H*_C_ reaching 2.4 kOe at RT due to enhanced pinning effects. At low temperature, a significant *H*_C_ increase (up to 3.5 kOe at 2 K) and a noticeable growth of *M*_S_ (10%) occurs mainly due to the reduction of thermally activated magnon population and the magnetic polarization of Pd by adjacent Co atoms, which strengthens the ferromagnetic coupling in the films.

The magnetization reversal in the porous films obeys mainly the Stoner–Wohlfarth rotational mechanism, with the double-step shape being characteristic of the corresponding magnetization curves due to the separate magnetization reversal of magnetically hard and soft regions. The ratio between these contributions is found to depend strongly on the film morphology.

A comparative analysis of the field dependences of magnetoresistance MR(*H*) and magnetization *M*(*H*) reveals the effect of spin waves propagation in both continuous and porous films and allows an identification of the dominant mechanism of magnetoresistance as coming from the electron scattering on magnons. The pronounced effect of spin waves in the films studied is consistent with a substantial intermixing of Co and Pd layers accompanied by the formation of CoPd ferromagnetic alloy. An increased negative MR effect and higher Bloch constant observed for the porous Co/Pd MLs, as compared to the continuous counterpart, indicate a strengthening of spin wave effects in the porous system. The effect is possibly related to their propagation in the undulated film, additionally modified with a quite regular array of pores.

## Methods

Templates of nanoporous TiO_2_ were fabricated by anodization of Ti film in 0.3% ammonium fluoride solution in ethylene glycol with 2 vol% of water at low temperature of electrolyte^24^. Two types of Ti films were used for anodization—(1) Ti foil with the thickness of 50 μm and (2) Ti film of 0.3 μm deposited onto Si wafer^46^. The anodization voltage was linearly increased from zero to 45–60 V with the rate of 1 V/s and then kept constant for the total anodization time, which was no longer than 35 min. The end of the anodization process was defined as the drop of the anodic current density below 30% of its maximum value^24,33,46^. Subsequent ion-plasma etching (Ar) was applied for additional smoothening of the surface relief. The etching time was varied from 60 min for TiO_2_ on Ti foil^46^ to 100 min for the templates fabricated over Si wafers^33,46^.

Co/Pd multilayers with nominal composition of Ta_5 nm_/Pd_15 nm_/[Co_0.5 nm_/Pd_1.0 nm_]_x5_/Pd_3 nm_/Ta_5 nm_ were deposited on anodized TiO_2_ templates. Continuous MLs of the same composition were also deposited on Si (Si/SiO_2_) wafers to serve as reference samples. All multilayers were fabricated using an ultra-high vacuum magnetron sputtering system (AJA International, Inc., USA) according to the procedure described previously^13,33,46^. The bilayers of Pd/Ta and Ta/Pd were used as seed and capping layers for promoting the (111) texture and for preventing the oxidation of the multilayers, respectively. The layer thicknesses were determined from the deposition time and calibrated deposition rates.

Surface morphology and cross-sectional microstructure of the templates and the films deposited on them was analyzed using a HITACHI S-4800 scanning electron microscope (SEM) at a voltage of 15 kV. The structures and phase compositions of the Co/Pd films were examined by X-ray diffraction (XRD) using an Empyrean PANalytical diffractometer with Cu Ka radiation (λ = 0.15418 nm). Experimental data were collected at a grazing incidence of 5° with respect to the sample surface, with the detector scanning the 2θ space from 10° to 120°. The experimental data were analyzed with HighScore Plus software and fitted with the FullProf program^47^ based on the Rietveld method.

The magnetic properties of the continuous and porous Co/Pd films were characterized using an alternating gradient magnetometer (AGM) and the vibrating sample magnetometer (VSM) option of a *Quantum Design* Physical Property Measurement System (PPMS) with external magnetic fields *H* up to 10 kOe applied along the film normal and up to 90 kOe applied in the film plane direction in the temperature *T* range of 2–300 K. The linear contribution of diamagnetic signal from the films substrates was subtracted from the experimental field dependences of magnetization *M*(*H*). Measurements of the field dependences of resistance *R*(*H*) were carried out using the resistivity option of the PPMS at *T* = 2–300 K. A linear press four-contact assembly was used for resistance *R* measurement using a square-wave excitation current with a frequency of 8.3 Hz applied parallel to the film surface. A magnetic field of up to 90 kOe was applied along the film normal.

## Data Availability

The data obtained and analyzed within this study are available from the corresponding author on reasonable request.
